# Socioeconomic determinants of stigmatization and HIV testing in Lesotho

**DOI:** 10.1080/09540121.2012.736937

**Published:** 2013-06-09

**Authors:** Lucia Corno, Damien de Walque

**Affiliations:** a Centre for Research and Analysis of Migration (CReAM), Department of Economics, University College London, London, UK; b The World Bank, Development Research Group, Washington DC, USA

**Keywords:** HIV/AIDS, stigma, Africa

## Abstract

HIV/AIDS stigmatizing attitudes and their consequences on preventative behaviors are among the most poorly understood aspects of the AIDS epidemic. This paper analyzes the socioeconomic determinants of discriminating attitudes toward people living with HIV and their implications on the likelihood of HIV testing. These effects are tested using the 2004 and 2009 Demographic and Health Surveys conducted in Lesotho, where HIV/AIDS is a pervasive problem. We find that HIV/AIDS stigmatizing attitudes are negatively associated with education and wealth and positively correlated with Catholic religion for women and traditional circumcision for men. The analysis also shows a negative association between stigmatizing beliefs and the probability of being tested for HIV.

## 1. Introduction

Despite substantial efforts to tackle HIV/AIDS, stigma and discrimination remain intractable challenges in addressing the epidemic. Previous literature investigated the determinants of stigma in African countries using population subsamples (Maughan-Brown, 2010; Nyblade & MacQuarrie, 2006; Ogden & Nyblade, 2005; Parker & Aggleton, 2003). Among others, Holzemer et al. (2009) document the levels of HIV stigma reported by persons living with HIV and nurses in Lesotho, Malawi, South Africa, Swaziland, and Tanzania. Further studies have also shown that stigma is a barrier to HIV testing (Day et al., 2003; Ehiri, Anyanwu, Donath, Kanu, & Jolly, 2005; Kalichman & Simbayi, 2003; Mahajan et al., 2008; Nyblade, Stangl, Weiss, & Ashburn, 2009; Obermeyer & Osborn, 2007; Weiser et al., 2006; Wolfe et al., 2008). For example, Meiberg, Bos, Onya, and Schaalma (2008) reported that the main barriers for testing were “fear of being stigmatised” and “fear of knowing their HIV-positive status” among a sample of South African students.

This paper describes the prevalence of HIV stigma and how stigma evolves over time using a nationally representative sample. Furthermore, we analyze the socioeconomic determinants of HIV/AIDS stigma and we investigate the association between stigma and the probability of HIV testing.

The analysis is conducted in Lesotho, a country with the third highest HIV prevalence in the world. In the 2004 Lesotho Demographic and Health Survey (LDHS), HIV prevalence is 23.2% for adults 15–49 years old, 26% for women aged 15–49, and 19% for men aged 15–59. In the 2009 LDHS, 23% of adults in Lesotho were infected with HIV (27% for women aged 15–49 and 18% for men aged 15–59).

## 2. Data and methods

The data used for this paper come from the 2004 and 2009 LDHS, conducted by the Ministry of Health and Social Welfare with the Bureau of Statistics and ORC Macro International (Lesotho Government and ORC Macro, 2005, 2010). The LDHS uses a representative sample of men and women of reproductive age in the 10 districts of Lesotho. Households were chosen for participating in the survey based on the following criteria: women aged 15–49 years were eligible to be interviewed; in every second household selected, all men 15–59 years were eligible. The final sample includes 20,833 respondents (9892 in 2004, 10,941 in 2009), 14,719 women and 6114 men. Average age is 28.1 years for women and 29.7 for men. Twenty-six percent of women live in urban areas compared to 23.3% of men, while 56.6% of women had some primary education (52.2% for men) and 41.1% had some secondary education (29.7% among men).

The survey includes five questions to measure attitudes toward HIV-infected people: (1) Would you buy vegetables from an HIV-positive vendor? (2) If a male teacher has the HIV virus, should he continue teaching in the school? (3) Same question for a female teacher; (4) If a family member got infected with HIV, would you want it to remain a secret? (5) If a relative became sick with HIV, would you be willing to care for her/him in your household?

Finally, the survey asks whether the respondent took an HIV test prior to the survey.

We first analyze the determinants of HIV stigmatization for both genders using multivariate analysis. More specifically, we run linear regressions where the dependent variable is an aggregate index of stigmatization ranging from 0 to 5, where 0 means no stigmatization and 5 is the maximum, determined by summing up five indicators based on the above questions. We check the reliability of the stigmatization score through the Cronbach's (or alpha) coefficient. In all regressions we control for demographic characteristics like age, age squared, education, religion and household features, the number of durable goods held by the household (television, ratio, refrigerator, motorbike, car, bicycle, electricity), and rural or urban area.

Second, we show the association of stigmatizing attitudes with having had an HIV test. We estimate a probit model where the dependent variable is equal to 1 if the respondent took an HIV test prior to the survey, and 0 otherwise. The independent variable is the stigmatization score described above and we add the same socioeconomic controls as in the first analysis. In all specifications, we use Stata 11 as statistical software and include district and year indicators, and robust standard errors are corrected for the correlation of the residuals at the cluster level.

## 3. Results

Table 1 shows percentages of women and men who hold HIV/AIDS-related stigmatizing views by age and characteristics. Overall, 38.3% of respondents say that they would not buy vegetables from an HIV-positive vendor (missing observations: 7.44%). 35.4 percent believe that an HIV-positive female teacher should not continue teaching (35.6% in the case of a male teacher) (missing observations: 7.20%). 37.8 percent say that if a family member was HIV positive, they would want it to remain secret (missing observations: 8%) and a smaller fraction (10%) expresses unwillingness to care for an HIV-positive relative in their household (missing observations: 10.3%).

**Table 1.** Fraction of individuals expressing stigmatization attitudes, by background characteristic.

Background characteristics	% who would not buy vegetables from an HIV-positive vendor	% who think a HIV-positive female teacher should stop teaching	% who think a HIV-positive male teacher should stop teaching	% who would want to keep secret if a family member got HIV	% who are not willing to care for an HIV-positive family member	Observation
**Gender**						
Female	0.361	0.315	0.320	0.382	0.087	14,719
Male	0.439	0.433	0.437	0.368	0.142	6114
**Age group**						
15–19	0.409	0.382	0.386	0.348	0.145	5191
20–24	0.359	0.326	0.333	0.347	0.105	4151
25–29	0.345	0.309	0.314	0.384	0.094	3058
30–34	0.357	0.305	0.310	0.401	0.074	2443
35–39	0.369	0.335	0.341	0.418	0.075	2003
40–44	0.401	0.368	0.373	0.411	0.081	1745
45–49	0.416	0.373	0.381	0.408	0.078	1612
50–54^(a)^	0.517	0.521	0.522	0.412	0.106	327
55–59(_a_)	0.570	0.545	0.545	0.407	0.128	303
**Residence**						
Urban	0.256	0.188	0.193	0.356	0.071	5352
Rural	0.431	0.410	0.414	0.386	0.114	15,481
**Marital status**						
Never married	0.367	0.332	0.338	0.338	0.123	8014
Married/living together	0.392	0.355	0.359	0.403	0.091	10,465
Divorced/separated/widowed	0.405	0.381	0.387	0.403	0.086	2253
**Education**						
No education	0.652	0.666	0.672	0.383	0.193	1397
Primary education	0.483	0.460	0.465	0.397	0.121	11,546
Secondary/tertiary education	0.208	0.152	0.157	0.351	0.064	7871
**Wealth quintile**						
Lowest	0.497	0.561	0.564	0.494	0.356	3870
Second	0.499	0.471	0.478	0.491	0.323	4106
Middle	0.397	0.367	0.370	0.375	0.097	3898
Fourth	0.324	0.260	0.265	0.368	0.087	4252
Highest	0.234	0.169	0.174	0.335	0.077	4707
*Total*	0.383	0.354	0.355	0.378	0.103	20833

Note: (a) Sample of men only. Source: authors’ calculations from the LDHS 2004 and 2009.

In general, a higher percentage of males express stigmatizing beliefs, with the exception of secrecy about HIV-positive relatives. Younger and older respondents are more likely to show HIV discriminating views than respondents between 20 and 39 years old. The proportion of individuals who report stigmatizing ideas decreases with education attainment, wealth and in urban areas.

**Figure 1.**
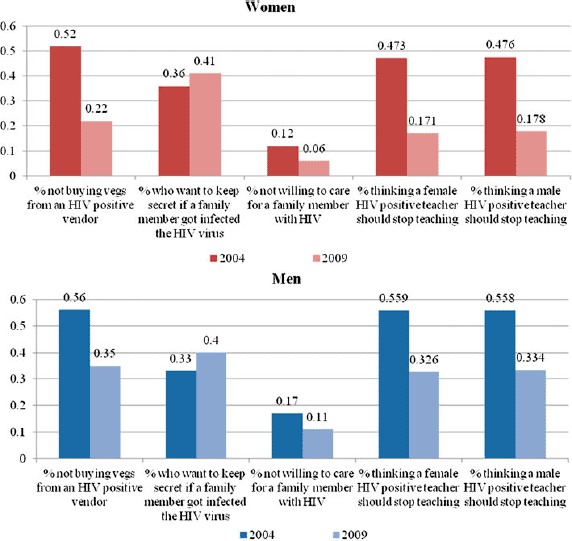
Trends in stigmatization attitudes toward those living with HIV/AIDS. Lesotho 2004–2009. Source: Lesotho Demographic and Health Survey 2004, 2009.

Figure 1 illustrates the change in stigma from 2004 to 2009 by gender. For both genders, the percentages who expressed discriminating attitudes have decreased significantly since 2004 for all attitudes, except for secrecy about an HIV-positive relative. This downward trend is stronger among women.

In Table 2, the dependent variable is an index from 0 to 5 summing up the stigmatization attitudes. The Cronbach's (or alpha) coefficient is 0.797 for the full sample, and 0.793 and 0.796 for women and men, respectively. According to the regression, the results from Table 1 are confirmed. Stigma first decreases and then increases with age (age squared is positive). Urban location, ownership of durable goods and education are negatively associated with stigma. There is also an association between religion and stigma: being Catholic is positively associated with stigma for women, but not statistically significant for men.

**Table 2.** Determinants of stigmatizing attitudes.

Dependent variable = Sum of HIV vendor + Stop teaching + keeping HIV secret + No care HIV-positive household member		
	Men (1)	Women (2)
Age	− 0.082^**^	–0.036^**^
	[0.011]	[0.008]
Age square	0.001^**^	0.000^**^
	[0.000]	[0.000]
Urban	− 0.265^**^	–0.221^**^
	[0.059]	[0.040]
Durable	− 0.372^**^	− 0.489^**^
	[0.064]	[0.112]
Primary education	− 1.218^**^	− 1.081^**^
	[0.076]	[0.121]
Secondary education	− 0.073^**^	− 0.098^**^
	[0.017]	[0.012]
Catholic	0.038	0.113^**^
	[0.046]	[0.041]
No religion	0.088	0.260
	[0.091]	[0.278]
Traditional circumcision	0.299^**^	
	[0.047]	
Year 2009 dummy	− 0.765^**^	− 0.616^**^
	[0.041]	[0.045]
District dummies	Yes	Yes
R-squared	0.26	0.22
Observations	5303	13,080

Notes: Table reports OLS coefficients. Robust standard errors in brackets. Estimates weighted with sample weights. The omitted category for education is “No education” and the omitted category for religion is “Protestant”. The stigmatization index is computed by summing up the answers to these five indicators: 1. Would you buy vegetables from an HIV-positive vendor? 2. If a male teacher has the HIV virus, should he be allowed to continue teaching in the school? 3. If a female teacher has the HIV virus, should she be allowed to continue teaching in the school? 4. If a member of your family got infected with the virus that causes AIDS, would you want it to remain a secret or not? 5. If a relative of yours became sick with the virus that causes AIDS, would you be willing to care for her/him in your own household?

* Significant at 5%; **Significant at 1%.

Men who have had traditional circumcisions are more likely to express stigmatizing attitudes.^1^

The 2004 LDHS shows that 14.5% of the population had been tested for HIV before the survey (11% for men, 15.8% for women), but that percentage sharply increases in the 2009 LDHS to 42.7% among men and 68.1 among women

Table 3 shows the results of bivariate and multivariate regressions analyzing the association between having had an HIV test before the survey with holding stigmatizing views, as measured by the sum of the five stigma indicators. According to the baseline estimates (columns 1 and 3), both men and women holding stigmatizing beliefs are significantly less likely to have been tested. Those results are robust to the inclusion of additional controls (columns 2 and 4).^2^

**Table 3.** Probability of having had an HIV test.

Dependent variable = 1 if already HIV tested prior the survey				
	Males		Females	
	(1)	(2)	(3)	(4)
Sum of all the STIGMA indicators	–0.040^**^	− 0.024^**^	− 0.029^**^	–0.027^**^
	[0.005]	[0.006]	[0.004]	[0.005]
Age		0.042^**^		0.088^**^
		[0.003]		[0.004]
Age square		− 0.000^**^		–0.001^**^
		[0.000]		[0.000]
Urban		− 0.003		–0.027
		[0.019]		[0.016]
Primary education		0.013		0.037
		[0.021]		[0.047]
Secondary education		0.094^**^		0.041
		[0.028]		[0.048]
Durables		0.003		–0.007
		[0.006]		[0.005]
Catholic		− 0.035^*^		0.001
		[0.014]		[0.017]
No religion		− 0.060^*^		0.026
		[0.028]		[0.111]
Male circumcision		− 0.013
		[0.016]
Year 2009 dummy	0.297^**^	0.307^**^	0.545^**^	0.572^**^
	[0.014]	[0.014]	[0.010]	[0.011]
District dummies	Yes	Yes	Yes	Yes
Observations	5343	5303	13,147	13,080

Notes: Table reports marginal probit coefficients. Robust standard errors in brackets adjusted for the correlation at the village level. Estimates weighted with sample weights. Table reports only the statistically significant coefficients. Additional covariates include secondary education, durable goods, and traditional circumcision. The stigmatization index is computed by summing the answers to these five indicators: 1. Would you buy vegetables from an HIV-positive vendor? 2. If a male teacher has the HIV virus, should he be allowed to continue teaching in the school? 3. If a female teacher has the HIV virus, should she be allowed to continue teaching in the school? 4. If a member of your family got infected with the virus that causes AIDS, would you want it to remain a secret or not? 5. If a relative of yours became sick with the virus that causes AIDS, would you be willing to care for her/him in your own household?

* Significant at 5%; **significant at 1%.

## 4. Conclusions and discussion

The determinants of HIV/AIDS-related stigma we identified in Lesotho may serve to guide future interventions and policies aimed at curbing stigma and encouraging HIV testing: stigma is *negatively* associated with education, wealth, and age, and *positively* associated with Catholic religion for women and traditional circumcision for men.

Some have asserted that interventions at the individual level are not sufficient, but that they must be coupled with changes at the local and national levels to societal structures that reinforce stigma, including poverty, inequality, and gender disparities, through changes to the legal system, making education more accessible, and creating more equitable economic opportunities (Ehiri et al., 2005; Mahajan et al., 2008; Ogden & Nyblade, 2005; South, Roura, Zaba, Todd, & Urassa, 2011). This aligns with our findings that the poor and less educated are more likely to have stigmatizing beliefs. Public leaders setting the example of getting tested paired with mass media anti-stigma campaigns can be effective in reducing stigma and increasing testing (Kalichman & Simbayi, 2003; Wolfe et al., 2008).

Given our finding of the association of Catholic women with stigmatizing attitudes, it could be helpful for faith-based leaders to promote anti-stigma attitudes (South et al., 2011).

The main limitation of the paper is a potential endogeneity in the empirical estimates: the indicators used to proxy HIV/AIDS stigmatization behaviors are not completely exogenous. Some omitted variables might influence both HIV-related stigma and the likelihood of being tested. Reverse causality is also a potential concern: being tested might help to diminish stigma. The estimated coefficients should therefore be interpreted with caution and not necessarily causally.
